# Ultrasonographic perithyroidal lymph node features predict thyroid autoantibody status: a clinical correlation study

**DOI:** 10.3389/fendo.2026.1783438

**Published:** 2026-06-12

**Authors:** Hongbing Xin, Zhengdong Fei, Hong Wang, Zheng Zhang, Bin Yang, Li Wang, Siqi Li, Lijuan Zhang, Yajun Zhou

**Affiliations:** 1 Department of Ultrasound, The Fourth Affiliated Hospital of Nanjing Medical University, Nanjing, China; 2 Department of Medical Ultrasound, Affiliated Hospital of Jiangsu University, Zhenjiang, Jiangsu, China; 3 Jinling Clinical Medical College, Nanjing Medical University, General Hospital of the Eastern Theater of the Chinese People’s Liberation Army, Nanjing, China

**Keywords:** autoantibody, early diagnosis, lymph nodes, thyroid, ultrasonography

## Abstract

**Background:**

Ultrasonographic diagnosis of thyroid diseases has traditionally relied on changes in thyroid echogenicity, which may appear relatively late in the disease course. This study aims to explore an early diagnosis for thyroid diseases before clinical symptoms occur by investigating the surrounding lymph nodes.

**Methods:**

This study used a combined case-control and prospective design. Initially, a retrospective review was performed on patients who underwent thyroid ultrasound examination and autoantibody testing at our hospital between February 2024 and July 2025. Patients were divided into four groups based on serological results. Ultrasonographic parameters were recorded for each group. Intergroup data comparison was performed using chi-square test and correlation analysis was conducted. Ultimately, the diagnostic efficacy of lymph node manifestations in each group was analyzed among 100 randomly selected patients with abnormal thyroid echogenicity, and a receiver operating characteristic (ROC) curve was plotted.

**Results:**

The prospective analysis revealed that the area under the ROC curve (AUC) for diagnosing Hashimoto’s antibody positivity using ultrasound detection of prelaryngeal, left paratracheal, and right paratracheal lymph nodes were 0.904, 0.845, and 0.829, respectively. When combining all three lymph node sites, the AUC reached 0.887, indicating high diagnostic accuracy, particularly for prelaryngeal lymph node assessment.

**Conclusions:**

Ultrasonographic imaging of lymph nodes surrounding the thyroid gland is significantly associated with the presence of Hashimoto’s thyroiditis-specific autoantibodies. These findings suggest that perithyroidal lymph node features may serve as valuable imaging markers for predicting autoimmune thyroid disorders, particularly Hashimoto’s thyroiditis.

## 1 Introduction

In current clinical practice, the diagnosis of diffuse thyroid diseases relies primarily on three components: clinical manifestations, serological testing, and ultrasonographic evaluation (1). Although histopathological analysis remains the gold standard for definitive diagnosis, its invasive nature and procedural limitations restrict routine application. As a non-invasive alternative, ultrasonography offers high-resolution imaging that facilitates detailed assessment of thyroid parenchymal echogenicity and vascular characteristics, thereby supporting individualized treatment planning.

However, the intrinsic limitations of ultrasound—including operator dependency and constrained spatial resolution—may hinder the early detection of subtle structural or functional abnormalities, potentially leading to missed or delayed diagnoses. In contrast, serological markers such as thyroid-specific autoantibodies and thyroid function profiles provide sensitive and specific insights at both immunological and metabolic level (2), offering valuable complementary information.

To address these diagnostic challenges, this combined case-control and prospective study investigates the association between ultrasonographic characteristics of perithyroidal lymph nodes and thyroid autoantibody positivity. We aim to refine ultrasonographic diagnostic criteria and improve early detection of autoimmune thyroid disorders by exploring this potential correlation.

## 2 Materials and methods

### 2.1 Study design

**Figure 1:**
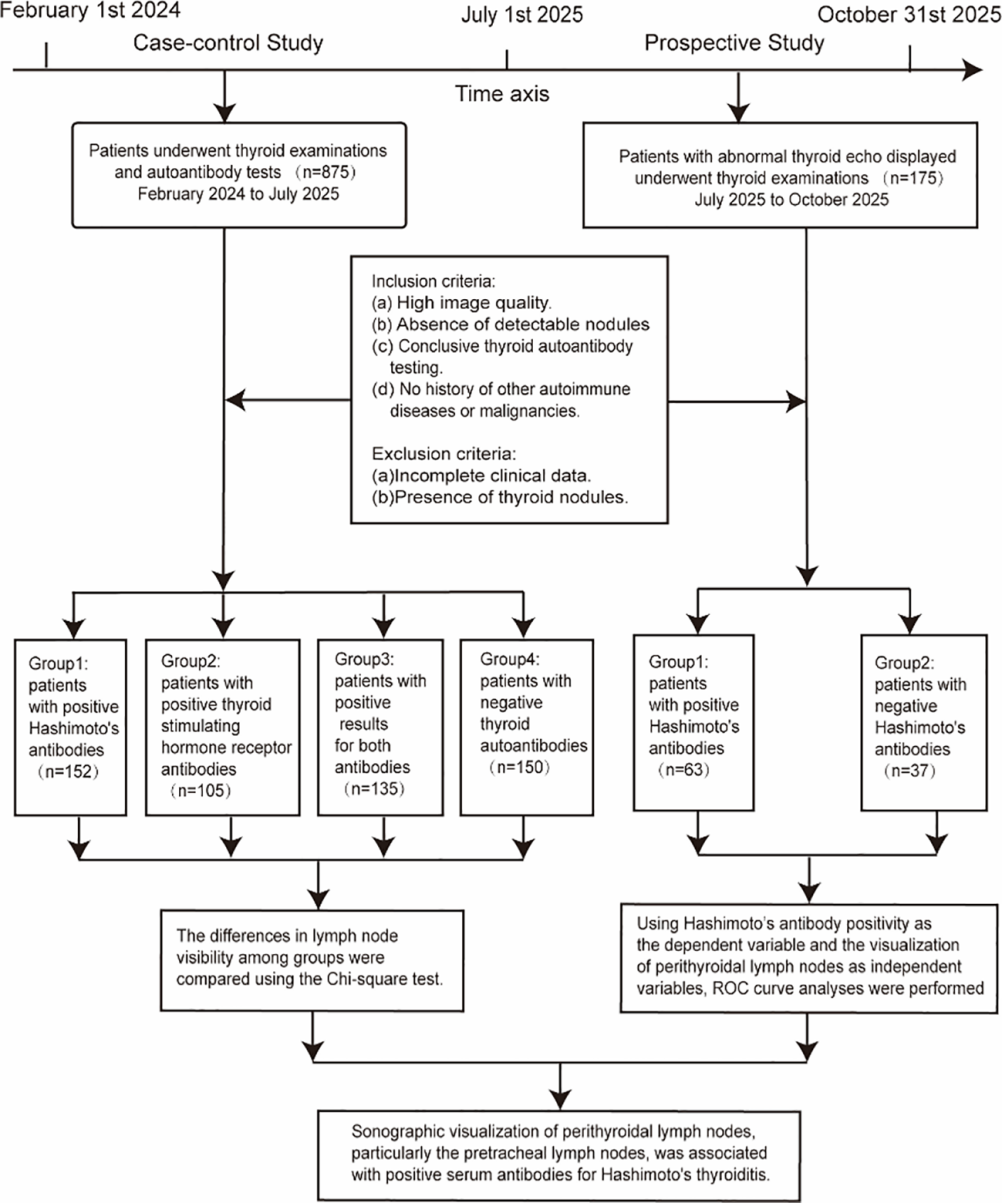
The flowchart of study procedure.

Clinical data of patients who underwent thyroid ultrasound examination and thyroid autoantibody testing at our hospital between February 2024 and July 2025 were included in this study. Patients were stratified into a control group and a study group according to the outcomes of thyroid autoantibody testing. The study group was further divided into three subgroups: Group 1 consisted of 152 patients with positive Hashimoto’s antibodies. Group 2 included 105 patients with positive thyroid stimulating hormone receptor antibodies. Group 3 comprised 135 patients with positive results for both antibodies. The control group (Group 4) was composed of 150 patients randomly selected from those with negative thyroid autoantibodies. The subjects of the prospective study were 100 randomly selected patients with abnormal thyroid echoes. Inclusion criteria for the study participants were as follows: (a) The ultrasound images were of high quality, ensuring clear visualization of the thyroid gland. (b) There were no detectable thyroid nodules during the ultrasound examination. (c) The patient has not been treated and the results of thyroid autoantibody testing are clearly positive. (d) Patients had no history of other autoimmune diseases or malignant tumors, as determined through comprehensive medical records review and relevant diagnostic evaluations. Exclusion criteria for the study participants were as follows: (a) The clinical data were incomplete. (b)Nodules were present in the thyroid gland (**Figure 1**).

This study was approved by the Ethics Committee of the Fourth Affiliated Hospital of Nanjing Medical University with the approval number of SFY20251123-K242.

### 2.2 Equipment and methods

#### 2.2.1 Ultrasound imaging protocol for the thyroid and perithyroidal lymph nodes

The patient is placed in a supine position on the examination bed to ensure complete exposure of the head and neck area. Any necklaces or other accessories that could potentially interfere with the examination are removed prior to the procedure. A Mindray R9 ultrasound diagnostic device, equipped with a 5–18 MHz high-frequency probe, is employed to perform a comprehensive and meticulous examination of the thyroid gland and the adjacent lymph nodes. During the examination, special emphasis is placed on evaluating the dimensions, shape, and echo characteristics of the thyroid gland. Additionally, the presence of any abnormal nodules is carefully noted. In the event that lymph nodes are detected in the vicinity of the thyroid gland, their precise location and dimensions are accurately recorded. In this study, thyroid ultrasound images were independently reviewed by two senior ultrasound physicians with extensive experience (associate chief physician level or above), both of whom were blinded to the pathological. In cases of disagreement, a third senior physician reviewed the images and a consensus decision was reached. All physicians interpreting the thyroid ultrasound images were blinded to the serological results and group assignments.

#### 2.2.2 Laboratory assessment for thyroid autoantibodies

Venous blood samples are collected from the research participants in the morning after an overnight fast. The collected blood is then subjected to centrifugation at 3500 revolutions per minute (rpm) for a duration of 10 minutes to isolate the serum. The serum specimens are subsequently analyzed using a Roche Cobas 801 fully automated electrochemiluminescence immunoassay analyzer.

### 2.3 Grouping basis

#### 2.3.1 Abnormal thyroid echo

**Figure 2:**
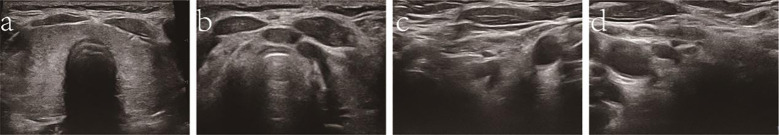
Ultrasound images of abnormal thyroid echo and surrounding lymph nodes. **(A)** abnormal thyroid echo; **(B)** prelaryngeal lymph nodes; **(C)** left paratracheal lymph nodes; **(D)** right paratracheal lymph nodes.

Normal thyroid ultrasound shows normal dimensions and shape, smooth capsule, homogeneous and fine parenchyma with equal or slightly higher echo. Abnormal thyroid echo may indicate an enlarged or reduced thyroid gland volume, an uneven capsule, decreased parenchymal echo, thickening, and unevenness. Scattered, fine linear or grid-like high echo septa can be observed (**Figure 2**).

#### 2.3.2 Lymph nodes around the thyroid

This includes the following three groups: a Prelaryngeal lymph nodes: Located in front of the larynx, directly in front of the cricothyroid membrane, the drainage area includes the isthmus of the thyroid and adjacent gland lobes, etc.; b Left and right paratracheal lymph nodes: Refers to those located below the thyroid and in the lateral grooves of the cervical segment of the trachea, closely adjacent to the tracheal wall, with the lateral boundary being the common carotid artery and the medial boundary being the midline, divided into left and right sides; The drainage area includes the left and right lateral lobes of the thyroid and other areas (**Figure 2**).

#### 2.3.3 Positive thyroid autoantibodies

Common autoantibodies of the thyroid include thyroid peroxidase antibody (TPOAb), thyroglobulin antibody (TGAb), and thyroid receptor antibody (TRAb). Positive expression of Hashimoto’s antibodies means that TPOAb ≥ 34 IU/L and/or TGAb ≥ 115 IU/L; TRAb ≥ 1.75 IU/L indicates positive expression of thyroid receptor antibody.

### 2.4 Statistical analysis

Statistical analysis was performed using SPSS 25.0 software. Count data were expressed as percentages (%), while measurement data were presented as mean ± standard deviation (x¯ ± s). Comparisons between the two groups were conducted using the χ2 test. Correlation was analyzed using the Spearman correlation coefficient. The receiver operating characteristic curve (ROC) was plotted to obtain the area under the curve (AUC), confidence interval (CI), sensitivity, and specificity. A difference was considered statistically significant if P < 0.05.

## 3 Results

### 3.1 General information

Hashimoto’s thyroiditis antibody-positive group (Group 1), thyroid-stimulating hormone receptor antibody (TRAb)-positive group (Group 2), dual antibody-positive group (Group 3), and autoantibody-negative control group (Group 4). A total of 152 patients were included in Group 1 (mean age: 45.6 ± 13.2 years; 48 males), 105 in Group 2 (mean age: 43.9 ± 9.9 years; 34 males), 135 in Group 3 (mean age: 44.0 ± 10.3 years; 44 males), and 150 in Group 4 (mean age: 44.5± 10.2 years; 52 males).

There were no statistically significant differences in age (*P* = 0.613) or gender distribution (*P* = 0.951) among the four groups. Detailed demographic data are presented in **Table 1**.

**Table 1:** General clinical data of patients.

	Group 1	Group 2	Group 3	Group 4	Total
Cases	152	105	135	150	542
Male	48 (31.6%)	34 (32.4%)	44 (32.6%)	52 (34.7%)	178
Female	104 (68.4%)	71 (67.6%)	91 (67.4%)	98 (65.3%)	364
Age (years)	45.6 ± 13.2	43.9 ± 9.9	44.0 ± 10.3	44.5 ± 10.2	

### 3.2 Thyroid echogenicity and perithyroidal lymph node findings by group

In Group 1 (n = 152), prelaryngeal lymph nodes were visualized in 145 patients, left paratracheal lymph nodes in 138 patients, right paratracheal lymph nodes in 130 patients, and thyroid echogenic abnormalities were detected in 125 patients.

In Group 2 (n = 105), prelaryngeal, left paratracheal, and right paratracheal lymph nodes were visualized in 9, 9, and 8 patients respectively, with 91 patients showing abnormal thyroid echogenicity.

In Group 3 (n = 135), prelaryngeal lymph nodes were observed in 108 patients, left paratracheal in 81, right paratracheal in 76, and abnormal thyroid echo was found in 116 patients.

In Group 4 (n = 150), only 8 patients had prelaryngeal lymph nodes visualized, 5 had left paratracheal, and 5 had right paratracheal lymph nodes visualized; abnormal thyroid echogenicity was detected in 22 patients. Detailed distributions are summarized in **Table 2**.

**Table 2:** Abnormalities of thyroid echoes and ultrasound imaging of perithyroidal lymph nodes in each group.

	Group 1	Group 2	Group 3	Group 4	Total
Total	152	105	135	150	542
Prelaryngeal lymph nodes					
Size (mm)	6.0×3.1	5.7×2.3	6.1×3.1	5.8×2.6	
Cases	145 (95.4%)	9 (8.6%)	108 (80.0%)	8 (5.3%)	270
Left Paratracheal lymph nodes					
Size (mm)	6.7×4.1	6.8×4.0	5.1×3.6	8.4×5.2	
Cases	138 (90.8%)	9 (8.6%)	81 (60.0%)	5 (3.3%)	233
Right paratracheal lymph nodes					
Size (mm)	6.3×3.9	5.9×4.4	7.1×4.3	6.2×3.6	
Cases	130 (85.5%)	8 (7.6%)	76 (56.3%)	5 (3.3%)	219
Abnormalities of thyroid echoes	125 (82.2%)	91 (86.7%)	116 (85.9%)	22 (14.7%)	354

In this study, the smallest detectable lymph nodes measure approximately 2 mm × 2 mm, while the largest measure about 10 mm × 6 mm.

### 3.3 Comparison between groups

**Figure 3:**
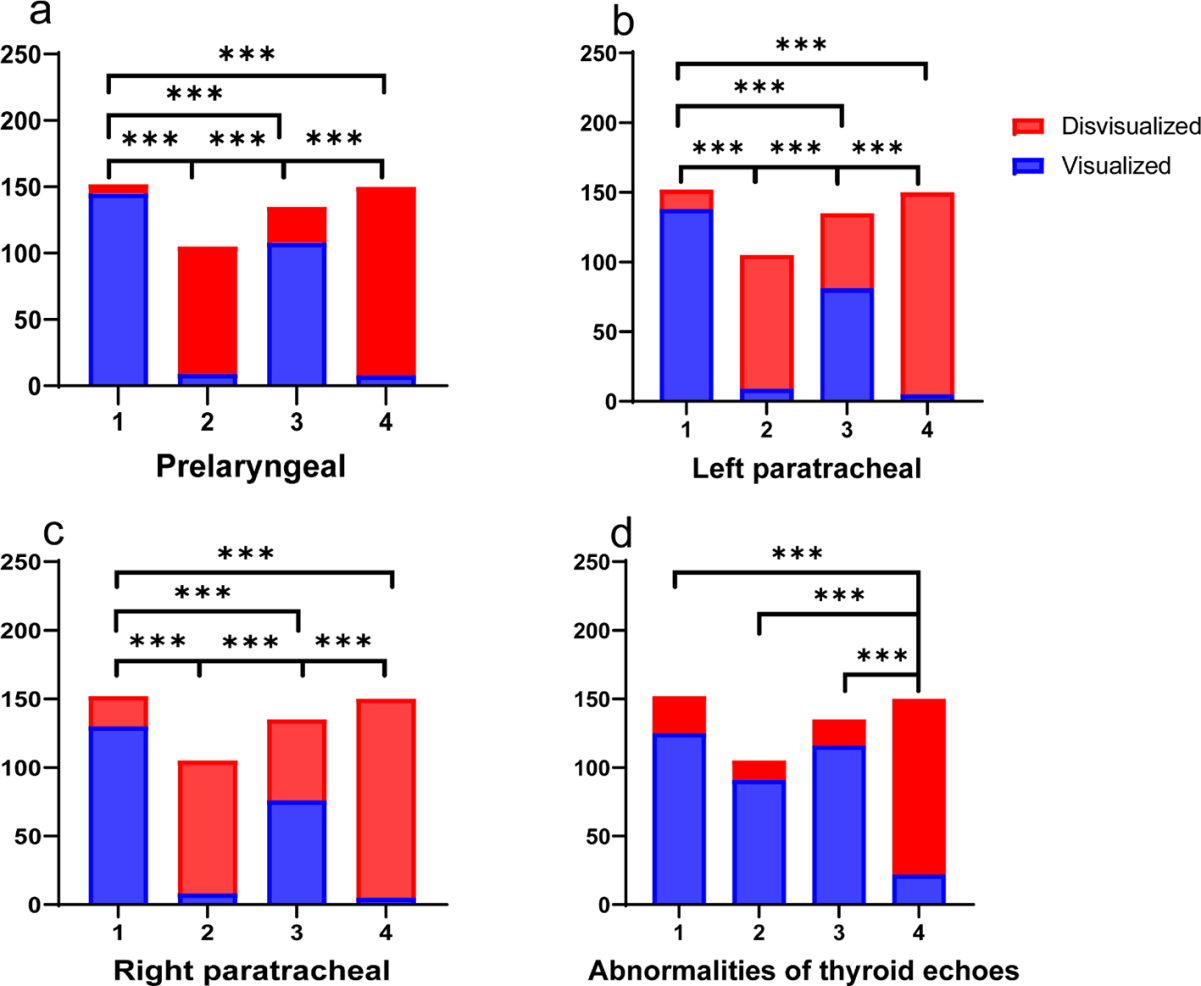
P values of thyroid echo abnormalities and ultrasound imaging of lymph nodes around the thyroid in among all groups. **(A)** Comparison of prelaryngeal lymph node visualization among the four groups; **(B)** Comparison of left paratracheal lymph node visualization among the four groups; **(C)** Comparison of right paratracheal lymph node visualization among the four groups; **(D)** Comparison of abnormal thyroid echogenicity among the four groups. ***P < 0.001.

Compared with the control group, both Group 1 and Group 3 exhibited statistically significant differences across all assessed indicators (all *P* < 0.001). Group 2 demonstrated a significant difference in the rate of thyroid echogenic abnormalities (*P* < 0.001); however, no significant differences were observed in the visualization of prelaryngeal, left paratracheal, or right paratracheal lymph nodes (*P* = 0.445, 0.094, and 0.153, respectively). These findings suggest a lack of statistical association between perithyroidal lymph node visualization and TRAb positivity. Detailed results are presented in **Figure 3**. Through statistical analysis, pairwise comparisons were conducted among the three groups. In terms of the lymph nodes around the thyroid displayed, all comparisons showed statistical significance (*P* < 0.001). The ultrasound imaging rate of the lymph nodes around the thyroid gland in group 1 and group 3 was significantly higher than that in group 2. There was no statistical difference in thyroid echo abnormalities (*P* > 0.005).

### 3.4 Correlation analysis between abnormal thyroid echo and the lymph nodes around the thyroid for positive Hashimoto’s antibody

The prelaryngeal lymph nodes showed the highest correlation, while abnormal thyroid echo showed the weakest correlation. Detailed results are presented in **Table 3**.

**Table 3:** Correlation analysis between abnormal thyroid echo and the perithyroidal lymph nodes for positive Hashimoto’s antibody.

Group	Rs
Prelaryngeal lymph nodes	0.833
Left Paratracheal lymph nodes	0.718
Right paratracheal lymph nodes	0.67
Abnormalities of thyroid echoes	0.452

### 3.5 Hashimoto’s antibodies and the perithyroidal lymph nodes manifestations in 100 cases of abnormal thyroid echo

In a prospective study of 100 patients with abnormal thyroid echo, 63 cases showed positive expression of Hashimoto’s antibodies and 37 cases showed negative expression; the display status of their surrounding lymph nodes is shown in **Table 4**.

**Table 4:** Hashimoto’s antibodies and the perithyroidal lymph nodes manifestations in 100 cases of abnormal thyroid echo.

Group	Positive expression(63)	Negative expression(37)
Prelaryngeal lymph nodes	56 (88.9%)	3 (8.1%)
Left paratracheal lymph nodes	52 (82.5%)	5 (13.5%)
Right paratracheal lymph nodes	50 (79.4%)	5 (13.5%)
Perithyroidal lymph nodes	59 (93.7%)	6 (16.2%)

### 3.6 Diagnostic efficacy of the lymph nodes around the thyroid for positive Hashimoto’s antibody

**Figure 4:**
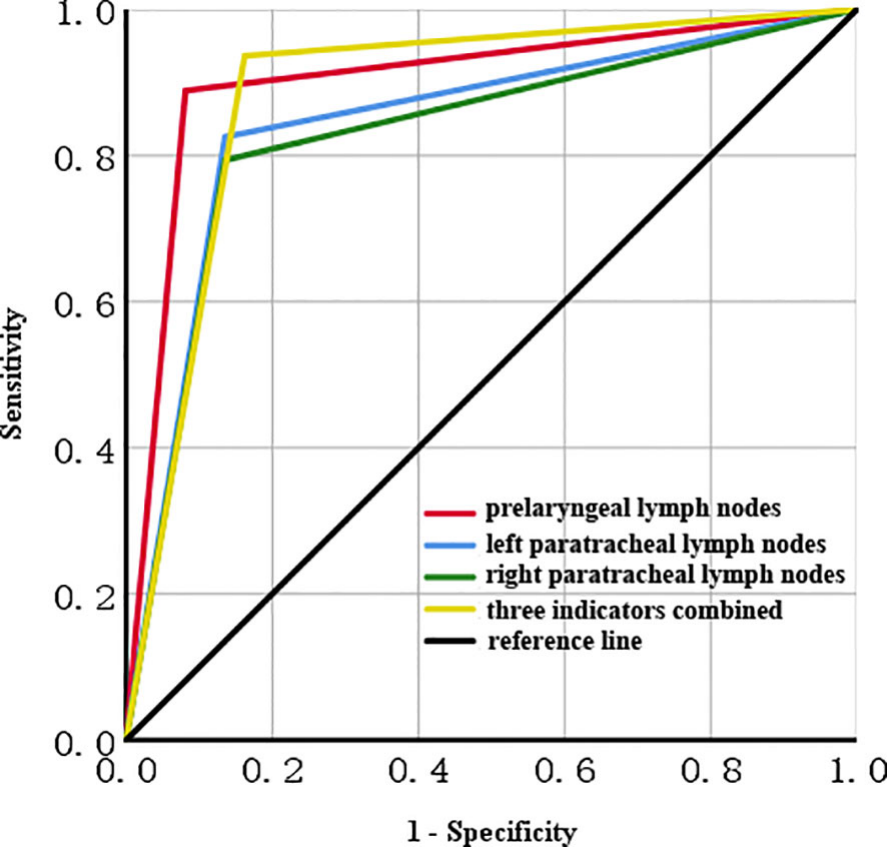
Diagnostic efficacy curve of the perithyroidal lymph nodes for the diagnosis of Hashimoto’s antibody positivity.

Using Hashimoto’s antibody positivity as the dependent variable and the visualization of perithyroidal lymph nodes as independent variables, ROC curve analyses were performed. The results demonstrated that the diagnostic efficacy of prelaryngeal lymph nodes was the highest, with an area under the curve of 0.904, and the sensitivity and specificity were 0.889 and 0.912 respectively. Details are provided in **Table 5**, **Figure 4**.

**Table 5:** Diagnostic efficacy of the perithyroidal lymph nodes for positive Hashimoto’s antibody.

	AUC	95%CI	Sensitivity	Specificity
Prelaryngeal lymph nodes	0.904	0.836-0.972	0.889	0.912
Left paratracheal lymph nodes	0.845	0.761-0.929	0.825	0.865
Right paratracheal lymph nodes	0.829	0.743-0.916	0.794	0.865
Three indicators combined	0.887	0.809-0.965	0.937	0.838

## 4 Discussion

Thyroid autoantigens primarily include thyroid peroxidase (TPO), thyroglobulin (TG), and thyroid-stimulating hormone (TSH) receptor, all of which play critical roles in thyroid hormone synthesis. TPOAb exerts its pathogenic effects by binding to TPO, thereby inhibiting enzymatic activity and mediating thyroid cell damage through antibody-dependent cell-mediated cytotoxicity (3). Studies have demonstrated that individuals testing positive for TPOAb have twice the risk of developing hyperthyroidism within six years compared to seronegative individuals (4). Recent research has demonstrated a close relationship between thyroid autoimmunity and metabolic disturbances (5). Moreover, TPOAb titers significantly correlate with both lymphocytic infiltration in the thyroid and the degree of hypoechogenicity observed on ultrasound. TGAb, another key autoantibody, triggers the deposition of immune complexes, complement activation, and the effect of antibody-dependent cell-mediated cytotoxicity by recognizing and binding to the key carrier Tg for thyroid hormone synthesis, thus destroying thyroid follicles and affecting thyroid function. TRAb comprises three subtypes: thyroid-stimulating antibody (TSAb), thyroid blocking antibody (TBAb), and thyroid binding inhibition antibody (TBI), which are the main indicators for Graves’ disease (GD) (6). TSAb mimics TSH by binding to TSHR, promoting excessive hormone production and follicular cell proliferation. In contrast, TBAb and TBI interfere with TSH signaling, leading to hypothyroidism or subclinical dysfunction (7).

Ultrasonographically, Hashimoto’s thyroiditis (HT) and GD are the most common autoimmune thyroid disorders associated with diffuse thyroid echogenic abnormalities. The global prevalence of HT is approximately 7.5%, with significant gender disparity—17.5% in women versus 6.0% in men—and geographic variation. The incidence varies in different regions (8), and it is also the main cause of hypothyroidism in iodine-sufficient areas (9). Pathologically, HT is characterized by progressive lymphocyte infiltration and follicular destruction, eventually culminating in fibrosis. Besides endocrine dysfunction, HT is also associated with elevated risks of malignancy. For example, the average incidence rates of thyroid cancer, breast cancer, and urogenital malignancies in HT patients are reported to be 25.01%, 1.40%, and 1.2%, respectively (10). GD, the leading cause of hyperthyroidism in iodine-sufficient populations, affects approximately 2% of women and 0.5% of men (11). Clinical manifestations include systemic hypermetabolism, cardiovascular overload, and characteristic ocular and dermatologic findings. In severe cases, it may be life-threatening, underscoring the necessity of early diagnosis and treatment to improve outcomes (12).

In this study, thyroid echogenicity abnormalities had the lowest correlation with positive Hashimoto’s antibody expression, with a coefficient of 0.452. Several factors may explain these findings. First, in early HT, follicular destruction may be minimal, resulting in detectable serological changes without overt sonographic abnormalities. In our cohort, 15.3% of patients fell into this category. Second, technical limitations such as equipment resolution may have impaired early structural detection.

Importantly, previous studies have identified perithyroidal lymph node visualization—particularly in tracheal regions—as a valuable indicator of autoimmune thyroiditis in adults, with diagnostic sensitivity and specificity of 93.4% and 74.5% (13). Kosiak et al. reported even higher accuracy in pediatric HT, with sensitivity of 98% and specificity of 100%, although GD patients were not included (14). In this study, the lowest AUC for lymph node-based diagnosis of Hashimoto antibody positivity was observed in the right paratracheal nodes (AUC = 0.829), with sensitivity and specificity of 0.794 and 0.865, respectively. These results suggest that perithyroidal lymph node enlargement may reflect immune activation and lymphatic drainage alterations secondary to autoimmunity.

TPOAb and TGAb are commonly referred to collectively as Hashimoto antibodies (15). Among HT patients, the positive rate of TPOAb exceeds 90%, while the positive rate of TGAb ranges from 60% to 80%. However, the presence of these antibodies alone does not confirm HT, as up to 70% - 80% of GD patients may also test positive for them (16). In this study, lymph node visualization was significantly lower in Group 2 (TRAb-positive) compared to Groups 1 and 3 (Hashimoto antibody–positive), which is consistent with the study by Xu Rong et al. (17). Possible explanations include generally lower titers of TPOAb and TGAb in GD and their limited lymphocytic infiltration, typically confined to the interlobular stroma, sparing thyroid follicles. Notably, the ultrasound imaging of perithyroidal lymph nodes in Group 3 was significantly lower than that in Group 1, indicating that Hashimoto antibody positivity—particularly in the absence of TRAb—may be a more specific marker for perithyroidal lymph node enlargement.

It is also important to consider other causes of perithyroidal lymphadenopathy in antibody-negative patients, such as subacute thyroiditis and thyroid cancer. Subacute thyroiditis typically involves both lobes, with blurred boundaries and hypoechoic features, although some normal parenchyma is usually preserved. In thyroid malignancies, metastatic nodes (commonly in levels III and VI) often present with irregular shapes, blurred margins, microcalcifications, cystic changes, and hypoechogenicity. In such cases, identification of the primary thyroid lesion is essential.

Several limitations should be noted in this study. First, we only assessed the association between perithyroidal lymph node findings on ultrasound and thyroid autoantibody positivity, and did not include fine-needle aspiration or histopathological confirmation of the thyroid gland. As a result, the relationship between these sonographic findings and specific thyroid disorders still needs further study. Second, patients with thyroid nodules were not included, which may limit the applicability of our results in routine clinical settings. Third, although the images were reviewed by experienced senior sonographers, some observer-related bias is still difficult to avoid completely. In addition, the prospective cohort was relatively small. Further studies with larger sample size and multicenter designs are needed to confirm the reliability and clinical value of these findings.

## 5 Conclusions

In summary, the visualization of perithyroidal lymph nodes—particularly prelaryngeal nodes—on ultrasound strongly suggests Hashimoto’s antibody positivity, even in the absence of overt thyroid parenchymal abnormalities. Such findings warrant prompt serologic testing, including thyroid function and autoantibody assays, to facilitate early diagnosis and prevent misdiagnosis or missed diagnoses.

## Data Availability

The raw data supporting the conclusions of this article will be made available by the authors, without undue reservation.
